# Clozapine as a Long-Term Therapeutic Choice: Longitudinal Analysis of Schizophrenia Symptoms in a Naturalistic Setting

**DOI:** 10.1093/schizbullopen/sgaf009

**Published:** 2025-05-30

**Authors:** Rachel K Scheinberg, Zhirui Fu, Laura Scott, Krista K Baker, Arlene Cuerdo, Lilian Zhong, Chloe Bethany, Malaka Harper, Leslie G Nucifora, Allison S Brandt, Russell L Margolis, Gayane Yenokyan, Frederick C Nucifora

**Affiliations:** Schizoaffective Disorder Precision Medicine Center of Excellence, Division of Neurobiology, Department of Psychiatry and Behavioral Sciences, Johns Hopkins University School of Medicine, Baltimore, MD 21287, United States; Department of Biostatistics, Johns Hopkins Bloomberg School of Public Health, Baltimore, MD 21205, United States; Department of Anesthesiology and Critical Care, Johns Hopkins University School of Medicine, Baltimore, MD 21287, United States; Psychiatry and Behavioral Medicine, Banner University Medical Center South, University of Arizona, Tucson, AZ 85713, United States; Johns Hopkins Bayview Medical Center, Baltimore, MD 21224, United States; Johns Hopkins Bayview Medical Center, Baltimore, MD 21224, United States; Johns Hopkins Bayview Medical Center, Baltimore, MD 21224, United States; Johns Hopkins Bayview Medical Center, Baltimore, MD 21224, United States; Schizoaffective Disorder Precision Medicine Center of Excellence, Division of Neurobiology, Department of Psychiatry and Behavioral Sciences, Johns Hopkins University School of Medicine, Baltimore, MD 21287, United States; Schizoaffective Disorder Precision Medicine Center of Excellence, Division of Neurobiology, Department of Psychiatry and Behavioral Sciences, Johns Hopkins University School of Medicine, Baltimore, MD 21287, United States; Schizoaffective Disorder Precision Medicine Center of Excellence, Division of Neurobiology, Department of Psychiatry and Behavioral Sciences, Johns Hopkins University School of Medicine, Baltimore, MD 21287, United States; Department of Neurology, Johns Hopkins University School of Medicine, Baltimore, MD 21224, United States; Schizoaffective Disorder Precision Medicine Center of Excellence, Division of Neurobiology, Department of Psychiatry and Behavioral Sciences, Johns Hopkins University School of Medicine, Baltimore, MD 21287, United States; Department of Biostatistics, Johns Hopkins Bloomberg School of Public Health, Baltimore, MD 21205, United States; Schizoaffective Disorder Precision Medicine Center of Excellence, Division of Neurobiology, Department of Psychiatry and Behavioral Sciences, Johns Hopkins University School of Medicine, Baltimore, MD 21287, United States

**Keywords:** treatment-resistant, ultra treatment-resistant, positive and negative syndrome scale

## Abstract

**Background and Hypothesis:**

Clozapine remains the gold standard for treatment-resistant schizophrenia (TRS), yet the time course of clinical response in naturalistic settings is not well characterized. We hypothesized that patients initiated on clozapine in an outpatient clinic would demonstrate measurable symptom reduction over time, including delayed response in a subset of patients.

**Study Design:**

We conducted a retrospective study of TRS patients (*N* = 26) newly initiated on clozapine at an outpatient clozapine clinic. Symptoms were assessed using the Positive and Negative Syndrome Scale (PANSS) at baseline and follow-up visits. Linear spline regression modeled PANSS trajectories over time. Response was defined as achieving either a ≥ 20% reduction in PANSS total score or a mild level of illness (PANSS score ≤ 58).

**Study Results:**

Patients demonstrated a mean 18.1-point reduction in PANSS total score during the first year of clozapine treatment, with significant improvements in positive and general psychopathology symptoms. Negative symptoms showed a modest, nonsignificant change. Overall, 20 patients (76.9%) achieved a ≥ 20% PANSS reduction, and 15 (57.7%) reached a mild symptom level. Six patients (23.1%) met response criteria only after 12 months of treatment.

**Conclusions:**

In this naturalistic study, clozapine was associated with substantial symptom improvement, particularly within the first year. A subset of patients demonstrated delayed but clinically meaningful response, supporting the continued use of clozapine beyond 12 months. These findings underscore the value of sustained treatment in TRS.

## Introduction

Schizophrenia, a severe and debilitating mental illness affecting approximately 1% of the population, is characterized by positive symptoms, negative symptoms, and cognitive impairment.^1–4^ Symptoms, course, and response to treatment are highly heterogeneous. Unfortunately, approximately 30% of patients do not respond to standard antipsychotic medicines.^5^ After 2 failed antipsychotic trials, these individuals are considered to have treatment-resistant schizophrenia (TRS).^6^

Clozapine is the only medication with a US Food and Drug Administration (FDA) approval for TRS.^7,8^ Multiple studies have demonstrated clozapine’s efficacy in reducing symptoms of TRS, though many of these investigations were of a short duration.^7,9–12^ Clozapine has additional benefits, including improved function and quality of life,^13^ and reduced risk of both suicide^14,15^ and tardive dyskinesia.^16^ Patients taking clozapine have a lower discontinuation rate, fewer hospitalizations, fewer adverse medical events, lower health care costs, and reduced mortality than comparable patients on other agents.^17–20^

Despite its unique benefits to patients, the healthcare system, and society, clozapine is greatly underutilized. While 30% of individuals with schizophrenia might benefit from clozapine, utilization may be as low as 4.4% in the United States.^8^ Even for patients who eventually receive treatment with clozapine, clozapine initiation is delayed by an average of 48 months after the second medication failure.^21^ Beyond the immediate consequences of untreated schizophrenia, delays in effective treatment are particularly concerning given the association of longer durations of untreated illness with worse prognosis.^22^

Contributing factors to the underutilization of clozapine include concerns about the risks of life-threatening side effects, particularly agranulocytosis and myocarditis,^23^ patient reluctance to undergo frequent blood draws,^24^ and the burden historically associated with the mandated clozapine Risk Evaluation and Mitigation Strategy (REMS) program,^24^ which required registration and monitoring by prescribers, pharmacies, and patients. Although the recent elimination of the REMS requirements by the FDA reduces some of the administrative complexity, significant clinical and logistical barriers to clozapine use remain. Nevertheless, for most patients with TRS, the benefits of clozapine use outweigh the risks.^25^

While the value of clozapine for TRS has been well-established,^13^ the relationship between duration of clozapine treatment and clinical improvement has not been thoroughly investigated, particularly in naturalistic clinical settings. In particular, it is unclear (1) if the clinical benefits of clozapine plateau, or, as in the response of bipolar patients to lithium,^26^ continue to accrue over time, and (2) if a subset of patients only respond to clozapine after a long duration of treatment. Establishing the pattern of response to clozapine may provide practical guidance for prescribers, and indirectly strengthen the case for clozapine utilization. The objective of the current study was therefore to assess the longitudinal symptom trajectories of patients on clozapine in a clinical setting, with the goal of determining the time course of symptom improvement after clozapine initiation, the extent of symptom improvement, and the particular symptom domains that improve. Importantly, we restricted the study to new starts, patients who were initiated on clozapine while under care in our clinic, ensuring that we had baseline PANSS scores on all participants and could follow them longitudinally from the start of treatment. This is not an acute treatment study, and the baseline PANSS scores do not reflect symptoms during an acute exacerbation, but rather represent the clinical status at the time of clozapine initiation in an outpatient setting.

## Methods

### Study Setting

The study protocol was approved by the Johns Hopkins Institutional Review Board. The study population consisted of patients with schizophrenia or schizoaffective disorder treated with clozapine at the Clozapine Clinic of the Johns Hopkins Bayview Community Psychiatry Program. The Clozapine Clinic treats approximately 100 patients from the Baltimore Metropolitan area. Enrollment includes patients transferring care from the Johns Hopkins Bayview Adult Schizophrenia Clinic at the initiation of clozapine treatment, and referrals from outside hospitals and clinics. Each patient is assigned to a psychiatrist (typically the clinic director, FCN) and a therapist (all credentialed as Licensed Clinical Professional Counselors or Certified Social Workers-Clinical). All patients receive cognitive behavioral therapy and/or supportive therapy for schizophrenia during weekly to monthly appointments with their therapist. A senior therapist oversees clozapine REMS issues in conjunction with the director of the clinic. Patients can access phlebotomy services and receive medicines at Johns Hopkins Bayview, or they can choose a laboratory and pharmacy of their convenience.

### Study Participants

Clinic patients were included in this study if they met DSM-5 criteria for schizophrenia or schizoaffective disorder, were prescribed clozapine, and had at least one Positive and Negative Syndrome Scale^27^ (PANSS) rating during clozapine treatment. Demographic and clinical data were collected from interviews with patients and their families or from the medical records. Only patients who were newly initiated on clozapine while under care in our clinic were included in this study. Baseline PANSS ratings were obtained immediately prior to clozapine initiation, and patients were followed longitudinally with subsequent PANSS assessments over the course of treatment. Time on clozapine was calculated by determining a start date from the medical record. Subjects were administered the PANSS at visits with their clinic therapist or psychiatrist. Clinic therapists and psychiatrists underwent PANSS certification, and inter-rater reliability was maintained through annual standardization meetings. Half the patients (*N* = 13) were rated by the same clinician during a regular clinical visit throughout the course of their treatment, while the other half of the patients had more than one clinician perform PANSS ratings at different time points due to clinician turnover.

Data collection began in February 2014 and ended in May 2023. All patients (*N* = 26) had a baseline PANSS completed before beginning clozapine, with the exception of 1 patient whose first PANSS rating occurred within one month of clozapine initiation before a therapeutic dose was reached. All patients were classified as having TRS based on failure to respond to at least 2 antipsychotic trials, each lasting more than 6 weeks. Patients were at least moderately ill, defined by a PANSS score > 58,^28^ except for 2 patients with PANSS scores of 58 or below, where the treating psychiatrist judged that clozapine treatment was warranted despite mild symptoms. A patient with a PANSS score of 58 or below is considered to be mildly ill or have a mild level of symptoms, based on the study by Leucht et al.,^28^ which used equipercentile linking between PANSS scores and Clinical Global Impression (CGI) scores to establish the clinical meaning of PANSS scores. Two outcome measures were used: (1) a PANSS total score declining to 58 or less, and (2) a PANSS total score decreasing by 20%, both for a minimum of one rating during the course of treatment. PANSS ratings were performed on average every 8.6 months while patients were enrolled in the clinic.

### Statistical Analysis

PANSS scores were modeled as a function of duration on clozapine using a linear spline regression model, with fixed knots at 3 and 6 years. We pooled all visits together to explore population-level effects, aiming to understand how certain factors impact the overall group of patients, rather than focusing on individual differences. By examining the population as a whole, we sought to identify trends and patterns that are consistent across the group, which may not be visible when analyzing each patient separately. Combining data from all visits provided a larger dataset, which enhanced statistical power and increased the reliability of our findings. Additionally, the pooled approach allowed us to model time-dependent trends, such as long-term disease progression or treatment efficacy over time.

To further investigate treatment effects during the early phase of clozapine use, we specifically focused on the first 3 years of treatment. For this subset of data, we employed a linear spline regression model with fixed knots at 1 and 2 years to assess changes in PANSS Total, Positive, Negative, and General scores, using change scores relative to baseline values.

To assess the proportion of patients who improved on clozapine (defined as achieving mild symptom levels or a ≥ 20% reduction in symptoms), we tracked PANSS scores at baseline, 6 months, 12 months, and beyond 12 months. At each time point, patient response was analyzed using both an absolute and a relative measure of improvement: (1) PANSS ≥ 58 or < 58, and (2) < 20% or ≥ 20% improvement from baseline PANSS score.

## Results

To explore the longitudinal benefit of clozapine, we examined PANSS data collected from patients at the Johns Hopkins Clozapine Clinic during their regular clinical care over a seven-and-a-half-year interval. The population included individuals with a baseline PANSS score who subsequently began clozapine while enrolled in the clinic.

### Study Population

As depicted in Table 1, the male to female ratio was 2.25:1. Despite the historical underutilization of clozapine among Black individuals,^29^ there were close to an equal number of Black and White participants (Black: 42.3%; White: 46.2%).

**Table 1.** Patient Characteristics

	(*N* = 26)
Age at first PANSS (SD)	32.33 (16.3)
Sex M (%)/F (%)	18 (69.23)/8 (30.77)
Race W (%)/B (%)/Other (%)	12 (46.15)/11 (42.31)/3(11.54)
Duration on clozapine (months) at last PANSS (SD)	43.27 (28.24)
Clinic visits with PANSS ratings (SD)	6.69 (3.5)
Mean interval between PANSS assessments (SD)	8.59 (8.06)

Age at first clinic visit with a PANSS score ranged from 12.8 to 68.6 years, x̄ = 32.3 years (standard deviation [SD]: 16.3). Initial PANSS score x̄ = 79.8, with scores ranging from 55 to 106.

A mean of 6.7 PANSS assessments were completed per patient (range: 2–14, SD: 3.5), with x̄ = 8.6 months (SD: 8.1) between each assessment. The mean duration on clozapine at the last completed PANSS was x̄ = 43.3 months (range: 3.6–89.8; SD: 28.2).

### Association of PANSS Scores With Duration of Clozapine Treatment

**Figure 1.**
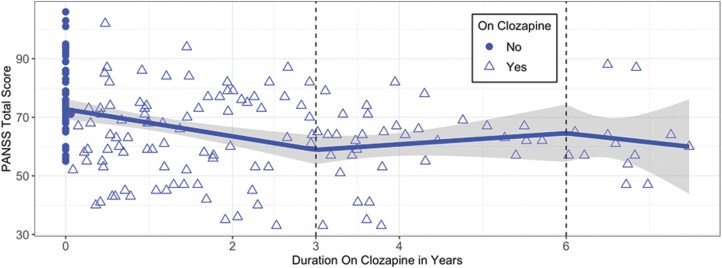
PANSS Total Score Improves During the First 3 Years of Treatment. Scatter Plot of All Visits and Fitted Line Between the Total PANSS Score and Duration on Clozapine (Unit: Years). The Line Represents the Linear Spline Regression Model With Knots at 3 and 6 Years. The Solid Circle Dots are PANSS Scores of Patients Before Starting Clozapine and the Hollow Triangles Show the Visits of Patients After Starting Clozapine. The Shaded Bands Around the Line are the 95% Confidence Intervals. The Model Shows a Significant Reduction in PANSS Scores Over the First 3 Years of Clozapine Treatment, Indicating Overall Symptom Improvement

Data were modeled using a linear spline model to explore the relationship between clozapine duration and PANSS scores across all available data points (Figure 1). Each point represents an individual PANSS score, and patients contributed an average of 3.4 scores between baseline and 3 years of clozapine treatment. To visualize individual trajectories, we generated spaghetti plots showing each participant’s PANSS scores over time on clozapine (Supplementary Data Content 1), corresponding to the data shown in Figure 1. Nearly three quarters (72.5%) of the data were collected within the first 3 years of clozapine use. PANSS scores improved by 4.6 points per year for the first 3 years of treatment (confidence interval [CI]: −6.9 to −2.4; *P* < .001). PANSS scores were available for all 26 patients between years 0–3 of clozapine use. However, PANSS scores were available for only 17 patients at 3–6 years of clozapine use and 5 patients at 6–7.5 years of clozapine use, reflecting the staggered initiation times of patients, with fewer individuals reaching later timepoints. Due to the limited data, symptom changes after more than 3 years of clozapine use are difficult to interpret.

Given the robust evidence of improvement in the first few years of clozapine treatment, we explored this time period in more detail.

### Improvement During the First 3 Years of Clozapine Treatment

**Figure 2.**
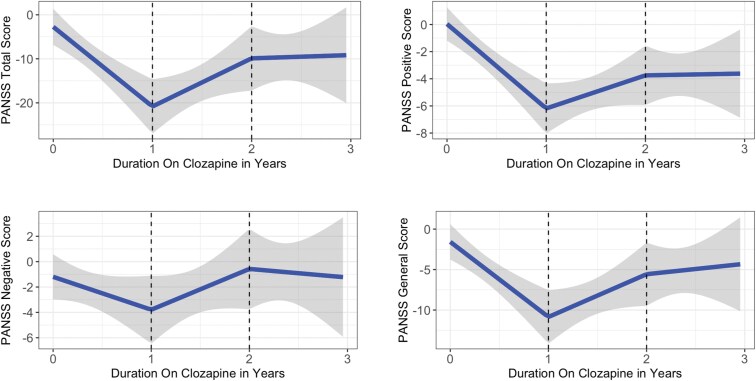
PANSS Scores Improve During the First Year of Treatment With Clozapine. The Fitted Lines Between the Total PANSS Score, Positive PANSS Score, Negative PANSS Score, General PANSS Score, and Duration on Clozapine (Unit: Years) for the New Start Cohort With Duration Between 0–3 years. The Lines Represent the Linear Spline Regression Model With Knots at 0, 1, and 2 Years. The Shaded Bands Around the Line are the 95% Confidence Intervals of the Fitted Lines. Statistically Significant Reductions Were Observed in Total, Positive, and General Scores During the First Year. Negative Symptoms Showed a Modest, Nonsignificant Improvement

The linear spine regression model assessing changes in PANSS relative to baseline values shows that most PANSS score improvements occurred during the first year on clozapine, with significant reductions in the total score as well as the positive and general psychopathology subscales (Figure 2, Table 2). Negative symptoms decreased modestly but non-significantly (Figure 2). After 1 year on clozapine, a slight increase in PANSS score was observed for all subdomains; however, PANSS total, positive, and general scores remained improved compared to baseline, whereas scores for the negative subdomain showed a gradual return toward baseline (see Discussion).

**Table 2.** First Year on Clozapine

PANSS subdomain	Slope (95% Conf. interval)	T-statistic	Effect size	*P*-value
Total score	−18.1 (−26.2, −10.0)	−4.41	−1.09	.000
Positive score	−6.2 (−8.7, −3.8)	−5.08	−1.21	.000
Negative score	−2.6 (−6.1, 0.9)	−1.46	−0.39	.147
General score	−9.3 (−13.6, −4.9)	−4.24	−1.06	.000

### Progression Toward Clinical Improvement: Time to Mild Symptoms or ≥ 20% PANSS Reduction

After 6 months on clozapine, 10 patients (38.5%) achieved a ≥ 20% improvement in PANSS scores, with 6 (23.1%) of these patients reaching a mild level of symptoms (PANSS ≤ 58) (Table 3). By 12 months, an additional 4 patients (15.4%) met the ≥ 20% improvement threshold, with PANSS scores of 1 patient (3.8%) declining to the mild category. Following 12 months of treatment, 6 more patients (23.1%) experienced a ≥ 20% improvement, all of whom also reached a mild level of symptoms. In total, 20 patients (76.9%) achieved a ≥ 20% improvement during treatment, and 15 patients (57.7%) reached a mild symptom level.

**Table 3.** Continued Improvement Over Time

Time point	Patients with mild symptoms (PANSS ≤ 58)	Percentage of total patients (*N* = 26)	Patients achieving 20% improvement	Percentage of total patients (*N* = 26)
By baseline	2	7.70%	N/A	N/A
By 6 months	6	23.10%	10	38.50%
By 12 months	1	3.80%	4	15.40%
After 12 months	6	23.10%	6	23.10%
Total	15	57.70%	20	76.90%

## Discussion

In this naturalistic study, we examined the course of symptoms in patients who began clozapine at the Johns Hopkins Bayview Community Psychiatry Clozapine Clinic after a baseline PANSS score was obtained. Most patients demonstrated significant and meaningful symptom improvement during the course of clozapine treatment. In total, 58% of patients improved to a mild level of symptoms (PANSS ≤ 58), 77% of patients improved by 20% or more, and 81% of patients met at least 1 of these criteria. Most of the improvement occurred in the first year of treatment, with significant reductions in both positive and general symptoms, while negative symptoms did not significantly change.

The findings are consistent, but expand upon the limited data on the value of long-term clozapine use for treating symptoms. A meta-analysis comparing clozapine to first-generation antipsychotics found clozapine superior, typically after a relatively short duration of use (6 weeks to 1 year).^30^ A second meta-analysis of randomized control trials of clozapine for TRS divided studies into short term (< 3 months of clozapine treatment), and long term (≥ 3 months of clozapine treatment). This analysis concluded that clozapine was superior to other antipsychotics in both the short and long term, but recommended changing to a different antipsychotic if there was no response to clozapine by 6 months.^31^ The Clinical Antipsychotic Trials of Intervention Effectiveness phase 2 study^10^ also demonstrated that clozapine was superior, based on length of time to clozapine discontinuation, compared to other second-generation antipsychotics.^10^ Here we demonstrate that for a subset of patients, there is potential for improvement after 12 months of clozapine, even if there had not been a significant response by 12 months. Further, patients in our clinic have successfully continued clozapine for years without discontinuation.

Our results are consistent with 2 long-term clozapine outcome studies. A retrospective 10-year study^32^ of TRS showed a greater improvement in symptoms with clozapine treatment compared to other antipsychotic medications. A prospective 14-year study^11^ showed significant symptomatic improvements from baseline or 6 months to study end as assessed with the Brief Psychiatric Rating Scale.^33^ Our data is consistent with these studies, but included more frequent PANSS ratings that enabled us to identify patients who responded to the extent that symptoms could be classified as mild.

There are several limitations to this study. The sample size is small, especially after 12 months. While the goal was to obtain PANSS scores for all patients every six months, the average interval between assessments was longer, and varied among patients, with some time points missing. This reflects the naturalistic setting of the study. Further, the successful use of the data, even though limited, demonstrates the feasibility and utility of obtaining objective data in the clinical setting of this patient population to help guide clinical treatment. Patients were classified as reaching a mild level of symptoms if their PANSS score was ≤ 58 at any assessment point, rather than classifying an adequate response solely on the final PANSS assessment. While not typical of most clinical trials, this approach enabled us to identify if a patient could ever respond to clozapine, and if so, how soon after clozapine initiation. Determining outcomes based on only the final symptom assessment risks underestimating the clozapine response.

The lack of continued improvement after the first year of clozapine treatment may reflect a plateau in treatment response or a limitation in the available data. Given the observational nature of the data and the absence of a comparison group, the extent to which clozapine contributed to the observed long-term outcomes cannot be determined with certainty. It is also possible that as patients stabilize and begin to reengage with life demands (work, relationships, independent living, etc.), emergent psychosocial stress could contribute to fluctuations in symptom severity or increased variability, reflected in PANSS scores.

We also note that while group-level PANSS scores plateaued after 12 months, nearly 1-quarter of patients achieved clinical response only after the first year. This suggests considerable heterogeneity in response trajectories, with some patients demonstrating delayed but meaningful improvements that may be obscured in group averages. More fundamentally, it is difficult to discern the extent to which improvement observed 12 months after the first PANSS assessment was a consequence of clozapine, or if therapy, access to psychosocial rehabilitation, other external factors, or variations in the intrinsic course of the illness, influenced clinical improvement. Finally, this study focused on symptom improvement as measured by the PANSS; the long-term impact of clozapine on functional, social, cognitive, or quality of life measures remains to be determined.

Overall, the data from this naturalistic study support the use of clozapine in TRS. A substantial portion of patients who did not improve after 12 months of clozapine treatment eventually improved with continued clozapine treatment, suggesting that in the absence of alternatives or major side effects, continuing clozapine past 12 months is clinically warranted even in patients not yet improving. Further, this study demonstrates the feasibility of initiating and maintaining clozapine for years in an outpatient setting, and the value of routine but rigorous use of rating scales during treatment.

## Supplementary Material

sgaf009_suppl_Supplementary_Data

## References

[1] Perälä J , Suvisaari J, Saarni SI, et al Lifetime prevalence of psychotic and bipolar I disorders in a general population. Arch Gen Psychiatry. 2007;64:19–28. https://doi.org/10.1001/archpsyc.64.1.19 17199051

[2] Saha S , Chant D, Welham J, McGrath J. A systematic review of the prevalence of schizophrenia. PLoS Med. 2005;2:e141. https://doi.org/10.1371/journal.pmed.0020141 15916472 PMC1140952

[3] Owen MJ , Sawa A, Mortensen PB. Schizophrenia. Lancet. 2016;388:86–97. https://doi.org/10.1016/S0140-6736(15)01121-6 26777917 PMC4940219

[4] American Psychiatric Association. Schizophrenia spectrum and other psychotic disorders. In: Diagnostic and Statistical Manual of Mental Disorders. 5th ed. Arlington, VA: American Psychiatric Publishing; 2013:100–103.

[5] Nucifora FC , Woznica E, Lee BJ, Cascella N, Sawa A. Treatment resistant schizophrenia: clinical, biological, and therapeutic perspectives. Neurobiol Dis. 2019;131:104257. https://doi.org/10.1016/j.nbd.2018.08.016 30170114 PMC6395548

[6] Howes OD , McCutcheon R, Agid O, et al Treatment-Resistant Schizophrenia: Treatment Response and Resistance in Psychosis (TRRIP) Working Group Consensus Guidelines on Diagnosis and Terminology. Am J Psychiatry. 2017;174:216–229. https://doi.org/10.1176/appi.ajp.2016.16050503 27919182 PMC6231547

[7] Meltzer HY. Treatment of the neuroleptic-nonresponsive schizophrenic patient. Schizophr Bull. 1992;18:515–542. https://doi.org/10.1093/schbul/18.3.515 1357741

[8] Meltzer HY. Clozapine: balancing safety with superior antipsychotic efficacy. Clin Schizophr Relat Psychoses. 2012;6:134–144. https://doi.org/10.3371/CSRP.6.3.5 23006238

[9] Rosenheck R , Cramer J, Xu W, et al A comparison of clozapine and haloperidol in hospitalized patients with refractory schizophrenia. Department of Veterans Affairs Cooperative Study Group on Clozapine in Refractory Schizophrenia. N Engl J Med. 1997;337:809–815. https://doi.org/10.1056/NEJM199709183371202 9295240

[10] McEvoy JP , Lieberman JA, Stroup TS, et al; CATIE Investigators. Effectiveness of clozapine versus olanzapine, quetiapine, and risperidone in patients with chronic schizophrenia who did not respond to prior atypical antipsychotic treatment. Am J Psychiatry. 2006;163:600–610. https://doi.org/10.1176/ajp.2006.163.4.600 16585434

[11] Lee MA , Cola P, Jayathilake K, Meltzer HY. Long-term outcome of clozapine in treatment-resistant schizophrenia. J Clin Psychopharmacol. 2023;43:211–219. https://doi.org/10.1097/JCP.0000000000001671 36975722

[12] Shankar G , Nate C. Positive and Negative Syndrome Scale as a long-term outcome measurement tool in patients receiving clozapine ODT—A Pilot Study. Pharm Pract (Granada). 2007;5:42–45. https://doi.org/10.4321/s1886-36552007000100007 25214917 PMC4155149

[13] Nucifora FC , Baker KK, Stricklin A, et al Better functional capacity and cognitive performance in clozapine responders compared to non-responders: a cross-sectional study. Schizophr Res. 2021;229:134–136. https://doi.org/10.1016/j.schres.2020.11.018 33234421

[14] Meltzer HY , Alphs L, Green AI, et al; International Suicide Prevention Trial Study Group. Clozapine treatment for suicidality in schizophrenia: International Suicide Prevention Trial (InterSePT). Arch Gen Psychiatry. 2003;60:82–91. https://doi.org/10.1001/archpsyc.60.1.82 12511175

[15] Taipale H , Lähteenvuo M, Tanskanen A, Mittendorfer-Rutz E, Tiihonen J. Comparative effectiveness of antipsychotics for risk of attempted or completed suicide among persons with schizophrenia. Schizophr Bull. 2021;47:23–30. https://doi.org/10.1093/schbul/sbaa111 33428766 PMC7824993

[16] Mentzel TQ , van der Snoek R, Lieverse R, et al Clozapine monotherapy as a treatment for antipsychotic-induced tardive dyskinesia: a meta-analysis. J Clin Psychiatry. 2018;79 :17r11852. https://doi.org/10.4088/JCP.17r11852 30257080

[17] Vanasse A , Blais L, Courteau J, et al Comparative effectiveness and safety of antipsychotic drugs in schizophrenia treatment: a real-world observational study. Acta Psychiatr Scand. 2016;134:374–384. https://doi.org/10.1111/acps.12621 27404582

[18] Stroup TS , Gerhard T, Crystal S, Huang C, Olfson M. Comparative effectiveness of clozapine and standard antipsychotic treatment in adults with schizophrenia. Am J Psychiatry. 2016;173:166–173. https://doi.org/10.1176/appi.ajp.2015.15030332 26541815

[19] Meltzer HY , Cola P, Way L, et al Cost effectiveness of clozapine in neuroleptic-resistant schizophrenia. Am J Psychiatry. 1993;150:1630–1638. https://doi.org/10.1176/ajp.150.11.1630 8105705

[20] Vermeulen JM , van Rooijen G, van de Kerkhof MPJ, Sutterland AL, Correll CU, de Haan L. Clozapine and long-term mortality risk in patients with schizophrenia: a systematic review and meta-analysis of studies lasting 1.1–12.5 years. Schizophr Bull. 2019;45:315–329. https://doi.org/10.1093/schbul/sby052 29697804 PMC6403051

[21] Howes OD , Vergunst F, Gee S, McGuire P, Kapur S, Taylor D. Adherence to treatment guidelines in clinical practice: study of antipsychotic treatment prior to clozapine initiation. Br J Psychiatry. 2012;201:481–485. https://doi.org/10.1192/bjp.bp.111.105833 22955007

[22] Edwards J , Maude D, McGorry PD, Harrigan SM, Cocks JT. Prolonged recovery in first-episode psychosis. Br J Psychiatry Suppl. 1998;172:107–116. 9764136

[23] Griffin JM , Woznica E, Gilotra NA, Nucifora FC. Clozapine-associated myocarditis: a protocol for monitoring upon clozapine initiation and recommendations for how to conduct a clozapine rechallenge. J Clin Psychopharmacol. 2021;41:180–185. https://doi.org/10.1097/JCP.0000000000001358 33587399

[24] Nucifora FC Jr , Mihaljevic M, Lee BJ, Sawa A. Clozapine as a model for Antipsychotic Development. *Neurotherapeutics*. 2017;14:750–761. https://doi.org/10.1007/s13311-017-0552-9 28653280 PMC5509641

[25] Fenton C , Kang C. Clozapine is the approved option in treatment-resistant schizophrenia and requires careful management. Drugs Ther Perspect. 2023;39:107–113. https://doi.org/10.1007/s40267-023-00982-6 36811119 PMC9936483

[26] Schou M , Juel-Nielsen N, Stromgren E, Voldby H. The treatment of manic psychoses by the administration of lithium salts. J Neurol Neurosurg Psychiatry. 1954;17:250–260. https://doi.org/10.1136/jnnp.17.4.250 13212414 PMC503195

[27] Kay SR , Fiszbein A, Opler LA. The positive and negative syndrome scale (PANSS) for schizophrenia. Schizophr Bull. 1987;13:261–276. https://doi.org/10.1093/schbul/13.2.261 3616518

[28] Leucht S , Kane JM, Kissling W, Hamann J, Etschel E, Engel RR. What does the PANSS mean? Schizophr Res. 2005;79:231–238. https://doi.org/10.1016/j.schres.2005.04.008 15982856

[29] Barry S , Jarskog LF, Xia K, Torpunuri RS, Wu X, Zeng X. Racial disparities in clozapine prescription patterns among patients with schizophrenia. Psychiatr Serv. 2024;75:733–739. https://doi.org/10.1176/appi.ps.20230226 38500451

[30] Moncrieff J. Clozapine v. conventional antipsychotic drugs for treatment-resistant schizophrenia: a re-examination. Br J Psychiatry. 2003;183:161–166. https://doi.org/10.1192/bjp.183.2.161 12893670

[31] Siskind D , McCartney L, Goldschlager R, Kisely S. Clozapine v. first- and second-generation antipsychotics in treatment-refractory schizophrenia: systematic review and meta-analysis. Br J Psychiatry. 2016;209:385–392. https://doi.org/10.1192/bjp.bp.115.177261 27388573

[32] Moreno-Sancho L , Juncal-Ruiz M, Vázquez-Bourgon J, et al Naturalistic study on the use of clozapine in the early phases of non-affective psychosis: a 10-year follow-up study in the PAFIP-10 cohort. J Psychiatr Res. 2022;153:292–299. https://doi.org/10.1016/j.jpsychires.2022.07.015 35878537

[33] Overall JE , Gorham DR. The brief psychiatric rating scale. Psychol Rep. 1962;10:799–812.

