# Supplementary material for: Clozapine as a Long-Term Therapeutic Choice: Longitudinal Analysis of Schizophrenia Symptoms in a Naturalistic Setting
Source: Schizophr Bull Open. 2025 May 30;6(1):sgaf009. doi: 10.1093/schizbullopen/sgaf009 (PMC12147019; doi:10.1093/schizbullopen/sgaf009)
Supplement: sgaf009_suppl_Supplementary_Data [file sgaf009_suppl_supplementary_data.docx]

**Supplementary Data Content 1:**


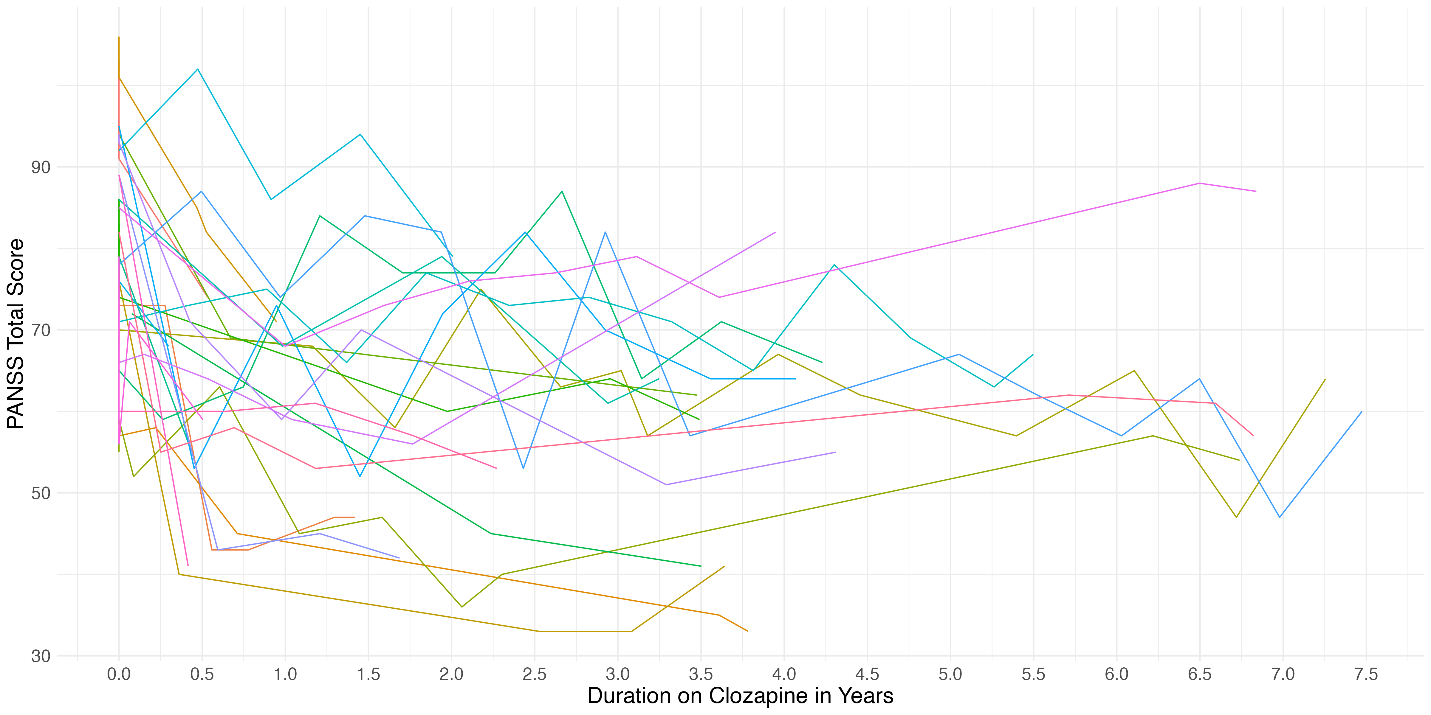


**Legend:** Individual PANSS scores overtime.

Spaghetti plot showing each participant’s PANSS scores over time on clozapine. Each line is an individual patient. The first score for each patient along the x-axis represents their baseline score before starting clozapine. This model shows there is variability in score trajectories while on clozapine.
